# The impact of nicotine dependence and smoking pattern changes on short- and long-term smoking cessation outcomes: The role of sociodemographic factors in a retrospective cohort study

**DOI:** 10.18332/tid/218297

**Published:** 2026-06-06

**Authors:** Birgül Deniz Doğan, Murat Doğan

**Affiliations:** 1 Department of Family Medicine, Faculty of Medicine, Ahi Evran University, Kirsehir, Türkiye

**Keywords:** smoking cessation, nicotine dependence, smoking patterns, Fagerström test, long-term outcomes

## Abstract

**INTRODUCTION:**

Smoking cessation success in real-world clinical settings remains limited, particularly among individuals with high levels of nicotine dependence. Although smoking cessation clinics are widely used, evidence regarding factors influencing both short- and long-term cessation outcomes is still limited. This study aimed to evaluate the association of nicotine dependence, smoking pattern changes, and sociodemographic characteristics with short-term (6-month) and long-term (12-month) smoking cessation outcomes.

**METHODS:**

This retrospective cohort study was conducted at the Smoking Cessation Outpatient Clinic of Kırşehir Ahi Evran University Faculty of Medicine Hospital. Adult patients who applied to the clinic between June 2023 and June 2024 and completed a 12-month follow-up between June 2024 and June 2025 were included. Sociodemographic characteristics, smoking-related variables, nicotine dependence assessed using the Fagerström test for nicotine dependence (FTND), and psychological characteristics were obtained retrospectively from medical records and self-reported follow-up assessments. Smoking cessation status at 6 and 12 months was analyzed using multivariable logistic regression analyses.

**RESULTS:**

At 12 months, 36.1% of participants achieved smoking cessation. Higher levels of nicotine dependence were consistently associated with lower odds of smoking cessation at both 6 and 12 months. Participants with very high nicotine dependence had significantly lower adjusted odds of smoking cessation at 12 months compared with those with very low dependence (adjusted odds ratio, AOR=0.078; 95% CI: 0.03–0.22). Changes in smoking patterns during follow-up, including both increases and decreases in cigarette consumption, were also independently associated with reduced long-term cessation success.

**CONCLUSIONS:**

Nicotine dependence severity and changes in smoking behavior are key determinants of long-term smoking cessation outcomes in real-world clinical settings. Identifying individuals at high risk of cessation failure may support more individualized follow-up strategies in smoking cessation clinics.

## INTRODUCTION

Tobacco use remains one of the major public health problems worldwide, causing millions of preventable deaths each year^1^. Numerous toxic and carcinogenic substances present in cigarette smoke have been shown to be directly associated with a wide range of chronic diseases, particularly cardiovascular diseases, chronic obstructive pulmonary disease (COPD), and various types of cancer^2^. Considering the substantial morbidity and mortality burden caused by these conditions, smoking cessation efforts are of critical importance for achieving individual health benefits and reducing the overall burden of disease at the population level^1,2^.

However, the process of smoking cessation is highly complex due to the strong addictive properties of nicotine^3^. By affecting reward and reinforcement pathways in the brain, nicotine triggers not only physiological dependence but also behavioral and psychological mechanisms that sustain smoking behavior^3^. These mechanisms contribute to the persistence of smoking and increase the risk of relapse. Consequently, unassisted quit attempts are often associated with low success rates, particularly among individuals with high levels of nicotine dependence, who frequently experience early failure during cessation attempts^4^. Existing evidence indicates that the severity of nicotine dependence is a key determinant of both short-term and long-term smoking cessation outcomes^3,4^.

The literature suggests that smoking cessation success is influenced not only by nicotine dependence but also by sociodemographic characteristics such as age, sex, education level, and socioeconomic status^5^. In addition, psychological conditions, including anxiety and depression, are known to be closely associated with smoking behavior and may complicate the cessation process^5,6^. The higher prevalence of smoking and lower cessation rates observed among individuals with psychiatric disorders further emphasize the clinical importance of these factors^6^. In this context, a detailed characterization of the clinical and psychological profiles of individuals attending smoking cessation clinics, provides an important framework for interpreting cessation outcomes.

Nevertheless, a substantial proportion of existing studies evaluating smoking cessation success tend to focus on a single time point or short-term outcomes, such as 30-day abstinence^7^. Given that relapse is common within the first year, short-term cessation success does not necessarily reflect sustained long-term abstinence. Real-world studies that simultaneously examine both short-term (6 month) and long-term (12 month) cessation outcomes and identify their associated determinants using multivariable analytical approaches, remain limited. This gap highlights an important deficiency in the current literature regarding predictors of long-term smoking abstinence.

Accordingly, the present study aims to examine the sociodemographic and clinical factors associated with short-term (6-month) and long-term (12-month) smoking cessation success among individuals attending a smoking cessation outpatient clinic, using a multivariable analytical framework. In particular, the study evaluates whether the level of nicotine dependence and changes in smoking behavior during the follow-up period represent independent determinants of long-term cessation failure.

## METHODS

### Study design and setting

This retrospective cohort study was conducted at the Smoking Cessation Outpatient Clinic of Kırşehir Ahi Evran University Faculty of Medicine Hospital. Adult patients who presented to the clinic between June 2023 and June 2024 were screened for eligibility. Follow-up assessments were completed between June 2024 and June 2025, enabling evaluation of smoking cessation status at both 6 and 12 months after the initial visit. Participants with missing outcome data or incomplete key variables were excluded from the final analysis.

### Study population

Patients aged ≥18 years who applied to the smoking cessation outpatient clinic during the defined inclusion period were eligible for the study. Individuals were included if they had complete baseline clinical data and documented smoking cessation status at both 6 and 12 months. Patients with missing baseline information, incomplete follow-up data, or irregular attendance that prevented outcome assessment, were excluded.

### Ethical considerations

The study protocol was approved by the Ethics Committee of Kırşehir Ahi Evran University Faculty of Medicine Hospital (Approval number: 2025-12/144; Date: 22 July 2025). Due to the retrospective nature of the study, the requirement for informed consent was waived. All procedures were conducted in accordance with the principles of the Declaration of Helsinki.

This study was reported in accordance with the Strengthening the Reporting of Observational Studies in Epidemiology (STROBE) guidelines, and the completed STROBE checklist is provided as the Supplementary file.

### Data collection and variable definitions

Data were collected retrospectively from medical records and the clinic database. Sociodemographic variables included age, sex, marital status (single, married, widowed), education level (illiterate, primary school, secondary school, high school, university degree), income status (income lower than expenditure, income equal to expenditure, income exceeding expenditure), and age group categories.

Smoking-related variables included age at smoking initiation, tobacco type, cumulative smoking exposure expressed as pack-years, previous intention to quit smoking (yes, no), and smoking pattern change. Cumulative smoking exposure was calculated as pack-years (packs per day × years of smoking). Smoking pattern change was evaluated by comparing daily cigarette consumption during follow-up with baseline levels, and was categorized as decreased, increased, or unchanged based on whether a meaningful reduction, increase, or no change in daily consumption was observed during the follow-up period.

Clinical characteristics included the presence of at least one physician-documented comorbid chronic disease identified from medical records. Psychological characteristics, including anxiety and depression levels, were assessed using the Hospital Anxiety and Depression Scale, and clinically significant anxiety and depression symptoms were defined using the commonly applied cutoff value of ≥8 for both HADS-A and HADS-D.

### Assessment of nicotine dependence

Nicotine dependence was assessed using the FTND, a widely used and practical instrument designed to evaluate the intensity of physical addiction to nicotine^8,9^. The FTND consists of six items that assess smoking-related behaviors reflecting dependence severity, including daily cigarette consumption, time to first cigarette after waking, and difficulty refraining from smoking in forbidden situations.

The total FTND score ranges from 0 to 10, with higher scores indicating greater nicotine dependence. In accordance with standard classifications, nicotine dependence levels were categorized as: very low (0–2), low (3–4), moderate (5), high (6–7), and very high (8–10).

The Turkish version of the FTND has previously been validated and shown to be a reliable assessment tool in the Turkish population. In particular, the study conducted by Uysal et al.^8^ demonstrated satisfactory psychometric properties of the FTND, supporting its use in both clinical and research settings in Turkey.

### Assessment of anxiety and depression

Psychological characteristics, including anxiety and depression levels, were assessed using the Hospital Anxiety and Depression Scale (HADS). The HADS is a self-report questionnaire consisting of 14 items, with 7 items assessing anxiety (HADS-A) and 7 items assessing depression (HADS-D). Each item is scored on a 4-point Likert scale ranging from 0 to 3, yielding subscale scores between 0 and 21 for both anxiety and depression, with higher scores indicating greater symptom severity.

The HADS was developed to assess anxiety and depression symptoms in medical populations and has been widely used in both clinical practice and research settings. The scale has demonstrated good reliability and validity across different populations and clinical conditions^10^.

### Outcome measures

Smoking cessation status was evaluated at 6 and 12 months after the initial clinic visit. Smoking cessation was defined as self-reported abstinence from smoking at each follow-up point and recorded as a binary outcome (quit vs not quit). Smoking status was obtained through face-to-face follow-up visits and telephone interviews and documented in the clinic follow-up records.

### Statistical analysis

Statistical analyses were performed using IBM SPSS Statistics version 29.0. Descriptive statistics are presented as means and standard deviations (SD) for continuous variables, and as frequencies and percentages for categorical variables. Associations between independent variables and smoking cessation status at 6 and 12 months were evaluated using chi-squared tests for categorical variables.

Variables showing a potential association with smoking cessation in univariate analyses were considered for further evaluation in multivariable models. Separate binary logistic regression analyses were performed for 6-month and 12-month cessation outcomes. Nicotine dependence level and smoking pattern change were entered into the models based on both clinical relevance and univariate findings. Anxiety and depression scores were not included in the final multivariable models due to their close relationship with nicotine dependence and the risk of multicollinearity. Collinearity diagnostics were examined using tolerance values and variance inflation factors in auxiliary models, and no concerning multicollinearity was identified.

Although anxiety and depression scores were assessed, these variables were not included in the multivariable models because of their close association with nicotine dependence and the potential risk of multicollinearity. Adjusted odds ratios (AORs) with 95% confidence intervals (CIs) are reported, and statistical significance was set at p<0.05.

Multicollinearity was assessed using collinearity diagnostics, including tolerance and variance inflation factor (VIF), in an auxiliary linear regression model. No problematic multicollinearity was detected among the predictors retained in the final models.

## RESULTS

A total of 407 patients who applied to the smoking cessation outpatient clinic and met the eligibility criteria were initially assessed. During follow-up, 108 patients were excluded because they had missing 6- or 12-month data and could not be reached by telephone, or did not attend scheduled follow-up visits. Consequently, the final analytic sample consisted of 299 participants with complete follow-up data. Most participants were male and middle-aged, and nearly three-quarters were married. Education level was predominantly at the high school level or higher. High or very high nicotine dependence was frequently observed, and more than half of the participants reported an increase in cigarette consumption during follow-up (Table 1).

**Table 1:** Sociodemographic and smoking-related characteristics of patients attending a smoking cessation outpatient clinic in Turkey, 2023–2024 (N=299)

*Characteristics*	*Categories*	*n (%)*
**Gender**	Male	175 (58.5)
	Female	124 (41.5)
**Age** (years)	18–24	25 (8.4)
	25–39	88 (29.4)
	40–54	108 (36.1)
	55–64	53 (17.7)
	≥65	25 (8.4)
**Marital status**	Single	67 (22.4)
	Married	224 (74.9)
	Widowed	8 (2.7)
**Education level**	Illiterate	5 (1.7)
	Primary school	67 (22.4)
	Secondary school	42 (14.0)
	High school	89 (29.8)
	University degree	96 (32.1)
**Income status**	Income < expenditure	88 (29.4)
	Income = expenditure	199 (66.6)
	Income > expenditure	12 (4.0)
**Age at smoking initiation** (years)	≤9	9 (3.0)
	10–14	89 (29.8)
	15–17	91 (30.4)
	≥18	110 (36.8)
**Smoking pattern change during follow-up**	Unchanged	105 (35.1)
	Decreased	35 (11.7)
	Increased	159 (53.2)
**Tobacco type**	Cigarettes (filtered/unfiltered)	261 (87.3)
	Other tobacco products	38 (12.7)
**Previous intention to quit smoking**	Yes	233 (77.9)
	No	66 (22.1)
**Nicotine dependence** (FTND categories)	Very low (0–2)	26 (8.7)
	Low (3–4)	44 (14.7)
	Moderate (5)	31 (10.4)
	High (6–7)	96 (32.1)
	Very high (8–10)	102 (34.1)

FTND: Fagerström test for nicotine dependence.

The distribution of nicotine dependence, anxiety, and depression scores is presented in Table 2. The mean FTND score indicated a moderate level of nicotine dependence among the study population. When categorized, a considerable proportion of participants were classified as having high or very high nicotine dependence.

**Table 2:** Clinical and psychological characteristics of patients attending a smoking cessation outpatient clinic in Turkey, 2023–2024 (N=299)

*Characteristics*	*Mean ± SD*	*Range*
**Age** (years)	44.69 ± 13.94	18–79
**Smoking exposure** (pack-years)	38.64 ± 24.81	0.5–180.0
**FTND** (score)	6.27 ± 2.34	0–10
**Anxiety score** (HADS-A)	7.84 ± 5.12	0–21
**Depression score** (HADS-D)	6.53 ± 4.71	0–21
**Age at smoking initiation** (years)	16.67 ± 4.97	6–45

FTND: Fagerström test for nicotine dependence. HADS: Hospital Anxiety and Depression Scale. HADS-A: Anxiety subscale. HADS-D: Depression subscale.

Regarding psychological characteristics, the majority of participants had low levels of anxiety and depression according to the Hospital Anxiety and Depression Scale. Most participants were classified as having low anxiety and depression levels based on HADS scores. Nevertheless, a substantial subgroup exceeded the established cutoff values for anxiety and depression, indicating the presence of clinically relevant psychological symptoms within the cohort.

Anxiety levels were not significantly associated with smoking cessation at either 6 or 12 months (Table 3). Depression level was also unrelated to cessation at 6 months; however, at 12 months, participants with lower depression scores achieved higher cessation rates compared with those with elevated scores (41.1% vs 28.0%, p=0.025).

**Table 3:** Factors associated with smoking cessation at 6 and 12 months among patients attending a smoking cessation outpatient clinic in Turkey, 2023–2024 (N=299)

*Variables*	*Categories*	*6-month cessation* *n (%)*	*p*	*12-month cessation* *n (%)*	p*
**Sociodemographic and clinical factors**					
**Gender**	Male	92 (52.6)	0.565	65 (37.1)	0.662
	Female	61 (49.2)		43 (34.7)	
**Marital status**	Single	27 (40.3)	0.116	19 (28.4)	0.097
	Married	121 (54.0)		88 (39.3)	
**Education level**	≤High school	104 (51.0)	0.966	74 (36.3)	0.778
	University	49 (51.0)		34 (35.4)	
**Income status**	Low	42 (47.7)	0.442	29 (33.0)	0.462
	Middle–High	111 (52.6)		79 (37.4)	
**Comorbid disease**	Present	78 (51.7)	0.865	51 (33.8)	0.394
	Absent	75 (50.7)		57 (38.5)	
**Smoking-related and psychological factors**					
**Smoking pattern change**	Unchanged	61 (58.1)	0.185	49 (46.7)	0.020
	Decreased	18 (51.4)		10 (28.6)	
	Increased	74 (46.5)		49 (30.8)	
**Nicotine dependence** (FTND)	Very low–Low	44 (61.1)	0.014	41 (56.9)	<0.001
	Moderate	19 (61.3)		16 (51.6)	
	High–Very high	90 (45.5)		51 (25.8)	
**Anxiety level** (HADS-A)	Low (<8)	80 (50.0)	0.572	60 (37.5)	0.660
	High (≥8)	73 (53.3)		48 (35.0)	
**Depression level** (HADS-D)	Low (<8)	105 (55.3)	0.085	78 (41.1)	0.025
	High (≥8)	48 (44.9)		30 (28.0)	

Statistically significant p values are shown in bold. FTND: Fagerström test for nicotine dependence. HADS: Hospital Anxiety and Depression Scale.

* P-values were obtained using the chi-squared test.

In multivariable logistic regression analyses, nicotine dependence remained independently associated with smoking cessation at both 6 and 12 months (Table 4). Compared with individuals with very low nicotine dependence, those with high and very high dependence had significantly lower odds of quitting at 6 months. This pattern became more evident at 12 months, with progressively lower odds of cessation observed across increasing dependence categories (AOR=0.29; 95% CI: 0.11–0.79; AOR=0.22; 95% CI: 0.08–0.59, respectively). This association was more pronounced at 12 months, showing a clear dose-response relationship across increasing nicotine dependence levels.

**Table 4:** Multivariable logistic regression analysis of factors associated with smoking cessation at 6 and 12 months among patients attending a smoking cessation outpatient clinic in Turkey, 2023–2024 (N=299)

*Variables*	*Categories*	*6 months* *AOR (95% CI)*	*p*	*12 months* *AOR (95% CI)*	*p*
**Smoking pattern change**	Unchanged (ref.)	1.00		1.00	
	Decreased	0.73 (0.33–1.61)	0.432	0.35 (0.14–0.89)	0.027
	Increased	0.67 (0.40–1.13)	0.131	0.53 (0.31–0.92)	0.025
**Nicotine dependence** (FTND)	Very low (ref.)	1.00		1.00	
	Low	0.36 (0.12–1.08)	0.068	0.25 (0.08–0.77)	0.015
	Moderate	0.46 (0.14–1.49)	0.195	0.27 (0.08–0.88)	0.030
	High	0.29 (0.11–0.79)	0.016	0.11 (0.04–0.31)	<0.001
	Very high	0.22 (0.08–0.59)	0.003	0.08 (0.03–0.22)	<0.001

AOR: adjusted odds ratios and 95% confidence intervals (CIs) were obtained using multivariable logistic regression analyses. Separate models were constructed for 6- and 12-month smoking cessation outcomes.

Smoking pattern change during follow-up was not independently associated with 6-month smoking cessation outcomes. However, at 12 months, both decreased and increased smoking patterns were associated with lower odds of smoking cessation compared with unchanged smoking behavior (Table 4).

## DISCUSSION

This study comprehensively examined the factors influencing short-term (6-month) and long-term (12-month) smoking cessation success among individuals attending a smoking cessation outpatient clinic, using real-world data. The findings confirm that the level of nicotine dependence is one of the strongest negative determinants of smoking cessation success at both time points^11^. In our multivariable analyses, increasing nicotine dependence levels were associated with a marked reduction in the odds of achieving smoking cessation at both 6 and 12 months^12^. In particular, participants with high and very high levels of nicotine dependence exhibited substantially lower cessation rates compared with individuals with lower dependence levels, clearly demonstrating the decisive clinical role of nicotine dependence severity in the smoking cessation process. This finding is consistent with the multivariable results showing a significant decline in cessation probability in both the short- and long-term as dependence severity increased, and it also contributes to the clinical interpretation of the generally low cessation rates observed in our study population.

When considering the underlying physiological and behavioral mechanisms, high nicotine dependence is well known to be associated with more severe withdrawal symptoms, stronger cravings, and reduced stress tolerance. These factors may increase the risk of relapse during follow-up and negatively affect long-term smoking cessation success. Moreover, individuals with high dependence have been reported to be more sensitive to environmental and social cues that trigger smoking behavior, further compromising the sustainability of quit attempts.

In comparison with previous studies, our findings are consistent with reports showing that high nicotine dependence is associated with frequent relapse and lower long-term cessation rates^13,14^. In this context, it has been emphasized that standard pharmacological treatment approaches may be insufficient for individuals with high levels of dependence, necessitating higher doses, longer treatment durations, or combined pharmacological therapies. Our results demonstrate that the negative impact of nicotine dependence is not limited to the short-term but persists during long-term follow-up, underscoring the need to individualize treatment intensity and follow-up duration according to dependence level and highlighting the importance of intensive pharmacological support strategies.

When compared with previous real-world studies, our results indicate that sociodemographic variables have limited predictive value for smoking cessation outcomes^15-17^. Another important finding of this study is that changes in cigarette consumption over time emerged as an independent determinant of long-term (12 month) smoking cessation success. Participants who either decreased or increased their cigarette consumption during follow-up had a lower odds of achieving long-term cessation compared with those whose smoking behavior remained unchanged, highlighting the critical role of behavioral stability in sustained smoking abstinence^18,19^. This finding suggests that quantitative changes in smoking alone do not guarantee long-term cessation and that, under real-world conditions, consistency in smoking behavior may be a stronger predictor of long-term success. Therefore, in smoking cessation clinics, it is important not only to assess nicotine dependence levels but also to evaluate recent smoking behavior trends and to plan individualized support strategies accordingly.

From a sociodemographic perspective, the lack of a significant association between variables such as sex, age, education level, income status, marital status and smoking cessation success at both short-term (6 months) and long-term (12 months) follow-up is noteworthy. This finding strengthens the hypothesis that smoking cessation success is largely determined by more intrinsic and clinical factors, such as nicotine dependence severity and behavioral characteristics, rather than sociodemographic attributes^20-22^. Although the literature presents heterogeneous findings regarding the role of sociodemographic factors, a systematic review based on real-world data and multivariable analyses frequently reports a diminished or absent predictive value of these variables^23^. Our results clearly indicate that smoking cessation interventions should prioritize modifiable clinical targets, particularly dependence severity and changes in smoking behavior, rather than sociodemographic characteristics^24^. This approach may enable more effective and efficient use of limited clinical resources.

Despite the high prevalence of at least one comorbid condition among participants, the absence of a significant association between comorbidity and smoking cessation success represents another noteworthy finding of this study^25^. This result supports the hypothesis that comorbid conditions may function more as motivational triggers rather than direct barriers to cessation^26^. Indeed, the most frequently reported reason for wanting to quit smoking among participants was the presence of a current illness, indicating that health concerns serve as a primary motivational factor. However, this health-driven motivation alone appears insufficient to ensure sustained cessation^27^. Particularly among individuals with high nicotine dependence, motivational approaches based solely on disease awareness may be inadequate, emphasizing the need for more intensive pharmacological and behavioral support to overcome physiological dependence^28^. In clinical practice, close monitoring of individuals with comorbid conditions and the implementation of individualized interventions tailored to dependence severity are critical for translating this motivational advantage into long-term cessation success^29^.

Although anxiety and depression levels were measured in this study, these variables were not directly included in the multivariable analyses. Nevertheless, the relatively high mean anxiety and depression scores observed suggest that psychological factors may play an important role in the smoking cessation process. The literature presents mixed results regarding the impact of anxiety and depression on cessation outcomes. While some studies report that higher anxiety and depression levels hinder cessation, others indicate that this association diminishes after accounting for nicotine dependence^20,28,30,31^. This suggests that psychological factors may influence cessation indirectly through their interaction with nicotine dependence, potentially exacerbating cravings and increasing relapse risk^32^. Future studies should further elucidate these indirect pathways and evaluate the effectiveness of tailored treatment approaches for individuals with psychological comorbidities.

### Strengths and limitations

The strengths of this study include the use of real-world data, a real world sample of 299 patients, the simultaneous evaluation of both 6- and 12-month smoking cessation outcomes, and the control of potential confounders through multivariable analyses.

Several limitations should be considered when interpreting these findings. Smoking cessation status was based on self-reported abstinence without biochemical verification, which may have resulted in misclassification. In addition, the retrospective design and reliance on medical records may have introduced recall bias. Restricting the analysis to individuals with a complete 12-month follow-up may also have led to selection bias. As this was a single-center study conducted in a smoking cessation outpatient clinic, the generalizability of the findings may be limited to similar real-world clinical settings.

## CONCLUSIONS

This study shows that nicotine dependence severity is a key determinant of both short-term and long-term smoking cessation outcomes. In addition, changes in smoking behavior during follow-up appear to influence long-term cessation success. These findings highlight the importance of tailoring cessation strategies according to dependence level and monitoring behavioral patterns during follow-up. Further prospective studies are needed to evaluate whether individualized treatment intensification can improve long-term abstinence, particularly among highly dependent smokers.

## Supplementary Material



## Data Availability

The data supporting this research are available from the authors on reasonable request.
