# Supplementary material for: The impact of nicotine dependence and smoking pattern changes on short- and long-term smoking cessation outcomes: The role of sociodemographic factors in a retrospective cohort study
Source: Tob Induc Dis. 2026 Jun 6;24:10.18332/tid/218297. doi: 10.18332/tid/218297 (PMC13248793; doi:10.18332/tid/218297)
Supplement: Supplementary file 1 [file TID-24-81-s1.pdf]

The STROBE reporting checklist

For checking that observational epidemiology research articles can be understood and used by everyone

Note

If you have not used a reporting guideline before, read about how and why to use them and check whether STROBE is the most applicable reporting guideline for your work.

Reporting guidelines are most useful when used early in research. When writing a manuscript or application, consider using the Full Guidance where you'll see explanations and examples for each item.

After writing, demonstrate adherence by completing this checklist:

1. Specify where each item is described (see Note 1).
2. Cite this checklist (See Note 2).
3. Include your completed checklist as a supplement when submitting to a journal so that future readers can use it to find information.

	Item Description	Location (or reason for not reporting)
**Title and abstract**		
1a. Indicate the study's design	Indicate the study's design with a commonly used term in the title or the abstract.	Title and Abstract; Methods – Study Design and Setting (first paragraph).
1b. Abstract	Provide in the abstract an informative and balanced summary of what was done and what was found.	Abstract (Introduction, Methods, Results, and Conclusion).
**Introduction**		
2. Background / rationale	Explain the scientific background and rationale for the investigation being reported.	Introduction, paragraphs 1–4.
3. Objectives	State specific objectives, including any prespecified hypotheses.	Introduction, final paragraph.
**Methods**		
4. Study design	Present key elements of study design early in the paper.	Methods – Study Design and Setting (first paragraph).
5. Setting	Describe the setting, locations, and relevant dates, including periods of recruitment, exposure, follow-up, and data collection.	Methods – Study Design and Setting.
6a. Eligibility criteria	**Cohort study:** Give the eligibility criteria, and the sources and methods of selection of participants.	Methods – Study Population; Inclusion Criteria; Exclusion

	Describe methods of follow-up. **Case-control study:** Give the eligibility criteria, and the sources and methods of case ascertainment and control selection. Give the rationale for the choice of cases and controls. **Cross-sectional study:** Give the eligibility criteria, and the sources and methods of selection of participants.	Criteria.
6b. Matching criteria	**Cohort study:** For matched studies, give matching criteria and number of exposed and unexposed. **Case-control study:** For matched studies, give matching criteria and the number of controls per case.	Not applicable; this was an unmatched retrospective observational cohort study.
7. Variables	Clearly define all outcomes, exposures, predictors, potential confounders, and effect modifiers. Give diagnostic criteria, if applicable.	Methods – Data Collection; Outcome Measures; Assessment of Nicotine Dependence; Assessment of Anxiety and Depression.
8. Data sources / measurement	For each variable of interest give sources of data and details of methods of assessment (measurement). Describe comparability of assessment methods if there is more than one group.	Methods – Data Collection; Assessment of Nicotine Dependence; Assessment of Anxiety and Depression.
9. Bias	Describe any efforts to address potential sources of bias.	Discussion – Strengths and Limitations.
10. Study size	Explain how the study size was arrived at.	Methods – Sample Size Considerations.
11. Quantitative variables	Explain how quantitative variables were handled in the analyses. If applicable, describe which groupings were chosen, and why.	Methods – Statistical Analysis.
12a. Statistical methods	Describe all statistical methods, including those used to control for confounding.	Methods – Statistical Analysis.
12b. Statistical methods – subgroups and interactions	Describe any methods used to examine subgroups and interactions.	No subgroup or interaction analyses were performed.
12c. Statistical methods – missing data	Explain how missing data were addressed.	Methods – Statistical Analysis.
12di. Statistical methods – loss to follow-up	**Cohort study:** If applicable, describe how loss to follow-up was addressed.	Methods – Study Population; Inclusion and Exclusion Criteria.
12dii. Statistical methods – matching cases and controls	**Case-control study:** If applicable, explain how matching of cases and controls was addressed.	Not applicable; this study did not use a case-control design or matching.
12diii. Statistical methods – sampling	**Cross-sectional study:** If applicable, describe analytical methods taking account of sampling	Not applicable; this was not a cross-sectional study with a

strategy	strategy.	sampling strategy.
12e. Statistical methods – sensitivity analyses	Describe any sensitivity analyses.	No sensitivity analyses were performed.
**Results**		
13a. Participant numbers	Report the numbers of individuals at each stage of the study—e.g., numbers potentially eligible, examined for eligibility, confirmed eligible, included in the study, completing follow-up, and analysed; Consider use of a flow diagram.	Results – first paragraph; Methods – Study Population.
13b. Participants – non-participation	Give reasons for non-participation at each stage.	Methods – Inclusion Criteria; Exclusion Criteria.
13c. Participants – flow diagram	Consider use of a flow diagram.	A flow diagram was not used; participant inclusion and exclusion are described in the Methods section.
14a. Descriptive data – participant characteristics	Give characteristics of study participants (e.g., demographic, clinical, social) and information on exposures and potential confounders. Present the information in a table.	Results – Tables 1, 2, and 3.
14b. Descriptive data – missing data	Indicate the number of participants with missing data for each variable of interest.	Methods – Statistical Analysis.
14c. Descriptive data – follow-up time	**Cohort study:** Summarise follow-up time—e.g., average and total amount.	Methods – Study Design and Setting; Outcome Measures.
15. Outcome data	**Cohort study:** Report numbers of outcome events or summary measures over time. **Case-control study:** Report numbers in each exposure category, or summary measures of exposure. **Cross-sectional study:** Report numbers of outcome events or summary measures.	Results – paragraphs reporting smoking cessation rates at 6 and 12 months; Tables 4 and 5.
16a. Main results	Give unadjusted estimates and, if applicable, confounder-adjusted estimates and their precision (e.g., 95% confidence intervals). Make clear which confounders were adjusted for and why they were included.	Results – Tables 4 and 5; corresponding Results text.
16b. Main results – category boundaries	Report category boundaries when continuous variables were categorised.	Methods – Assessment of Nicotine Dependence; Tables 2, 3, and 5.
16c. Main results – risk	If relevant, consider translating estimates of relative risk into absolute risk for a meaningful time period.	Not applicable; relative effect estimates (odds ratios) are reported without conversion to absolute risk.

17. Other analyses	Report other analyses done—e.g., analyses of subgroups and interactions, and sensitivity analyses.	No additional subgroup, interaction, or sensitivity analyses were performed.
**Discussion**		
18. Key results	Summarise key results with reference to study objectives.	Discussion – first and second paragraphs.
19. Limitations	Discuss limitations of the study, taking into account sources of potential bias or imprecision. Discuss both direction and magnitude of any potential bias.	Discussion – Strengths and Limitations paragraph.
20. Interpretation	Give a cautious overall interpretation considering objectives, limitations, multiplicity of analyses, results from similar studies, and other relevant evidence.	Discussion – middle paragraphs.
21. Generalisability	Discuss the generalisability (external validity) of the study results.	Discussion – final paragraph.
**Other information**		
22. Funding	Give the source of funding and the role of the funders for the present study and, if applicable, for the original study on which the present article is based.	Funding section.

© 2026 Deniz Doğan B. and Doğan M.
